# Supplementary material for: Polyketide synthase gene clusters in the ericoid mycorrhizal fungus Oidiodendron maius: genome-wide prediction, expression profiling, and functional characterization of knockout mutants
Source: Front Plant Sci. 2026 Sep 16;17:1924430. doi: 10.3389/fpls.2026.1924430 (PMC13623903; doi:10.3389/fpls.2026.1924430)
Supplement: Supplementary file 1 [file SupplementaryFile1.zip › Supplementary Materials/Supplementary Folder 1.zip!/Oidma1_AssemblyScaffolds_Repeatmasked/knownclusterblast/cluster55/jgi.p_Oidma1_184988_mibig_hits.html]

| MIBiG Protein | Description | MIBiG Cluster | MiBiG Product | % ID | % Coverage | BLAST Score | E-value |
| --- | --- | --- | --- | --- | --- | --- | --- |
| EAL89346.1 | extracellular\_dihydrogeodin\_oxidase/laccase,\_putative | BGC0001403 | Trypacidin\_biosynthetic\_gene\_cluster | 59 | 99.1666666667 | 767 | 9.1e-222 |
| AGO59042.1 | PtaE | BGC0000121 | Pestheic\_acid\_biosynthetic\_gene\_cluster | 55 | 85.6666666667 | 608 | 7e-174 |
| AGO59052.1 | PtaK | BGC0000121 | Pestheic\_acid\_biosynthetic\_gene\_cluster | 34 | 44.5 | 148 | 3.4e-35 |
